# Directional bio‐synthesis and bio‐transformation technology using mixed microbial culture

**DOI:** 10.1111/1751-7915.13924

**Published:** 2021-09-15

**Authors:** Hui Wang

**Affiliations:** ^1^ State Key Joint Laboratory on Environment Simulation and Pollution Control School of Environment Tsinghua University Beijing China

In the past 15 years since its inception, Microbial Biotechnology (MBT) has been a top journal in microbial technology. MBT is committed to promoting innovative, diverse and socially beneficial microbial technology research. It provides a platform for researchers to share innovative research and cutting‐edge ideas. Even minor technological advances will undoubtedly promote the application of microbial technology in the future.

From the perspective of nature and humans’ sustainable development, waste recycling and pollutant removal are two critical research areas. In the past 20 years, the application of biotechnology in these two aspects has made remarkable achievements. Since the breakthroughs of DNA sequencing technology in 1976 and its third generation in 2011, the rapid development of sequencing technology has dramatically promoted the detection capability of environmental micro‐organisms. It deepens people’s understanding of microbial metabolism and inter‐species relationship, thereby accelerating the application of microbial technology in the environmental field. Over the next 15 years, various genomic technologies and novel high‐throughput isolation and culture methods will be used to accelerate the exploration of our biosphere, which will further promote the application of microbial biotechnology.

Integrating synthetic biology and microbial technology, microbial synthesis technology has a broad market and economic benefits in environmental resource treatment. For instance, waste carbon sources are transformed by microbial synthesis to volatile fatty acid, medium‐chain fatty acid (represented by caproic acid), ethyl butyrate, polyhydroxyalkanoate (PHA) and other products, which are essential raw materials for medical treatment, food, cosmetics and biodegradable plastics (Donno Novelli *et al*., 2021). The microbial synthesis of PHA from urban waste organic matter provides the most effective way to reduce the global ‘white pollution’, representing the promising application of microbial synthesis as Greentech in solid waste recycling. Nowadays, the use of mixed microbial culture (MMC) to produce PHA has been well developed in laboratories and is increasingly explored at the pilot scale (Crognale *et al*., 2019; Conca *et al*., 2020). However, large‐scale industrial production is still limited, primarily due to low PHA accumulation and biomass concentration. The constraints are probably attributed to the competition in space and nutrients between microbial populations in MMC fermentation. Besides, the different utilization efficiency of carbon sources in the mixed substrate will affect the growth and PHA synthesis metabolism of different micro‐organisms. The maximum bacterial density for pure bacterial production on an industrial scale can reach 150 g l^‐1^ (Ryu *et al*., 2015). In contrast, the maximum cell concentration in MMC is usually < 10 g l^‐1^ (Moretto *et al*., 2020; Matos *et al*., 2021), indicating the lack of biomass which greatly limits volumetric productivity. Therefore, future research will focus on improving PHA yield, which will be the only way to promote the transition of PHA synthesis from pilot‐scale to industrial production. Research to improve PHA yield will primarily focus on optimizing the enrichment and accumulation process of MMC. In the industrial production of PHA synthesis by MMC, the balance between high PHA accumulation rate and high biomass of MMC must be maintained in the future. Furthermore, in the future, more industrial organic wastewater (brewing, papermaking, food and crude glycerol) will be used as an alternative carbon source for PHA production. Then, the use of high‐cost carbon sources and the cost of industrial organic wastewater treatment will be reduced, leading to sustainable and more market‐efficient industrial production (Nguyenhuynh *et al*., 2021).

The elimination/wide distribution of persistent environmental pollutants (POPs) on the earth is one of the most severe environmental problems. Among the POPs, petroleum hydrocarbons, polycyclic aromatic hydrocarbons (PAHs) and halogenated hydrocarbons are the major environmental pollutants that threaten human health because of their high hydrophobicity and high toxicity. Over 20 years of effort paved the significant development in research about aerobic PAHs‐degrading micro‐organisms. According to the incomplete statistics of previous studies, over 100 genera of bacteria were verified to grow with different PAHs as the sole carbon source under aerobic conditions, and the major aerobic PAHs biodegradation pathways, degradation genes and enzymes were intensively studied. Meanwhile, functional gene markers were further extended from the PAHs ‐hydrolating dioxygenase large subunit gene (*pahAc*) to trans‐o‐hydroxybenzylidenepyruvate hydratase‐aldolase gene (*pahE*) to detect the species and activity of aerobic PAHs‐degrading micro‐organisms in various environments. *pahE* led to much higher detection accuracy and more accurate correlation with PAHs degradation efficiency, facilitating water or soil remediation by biotechnology(Chengyue *et al*., 2018). Recently, anaerobic biodegradation of organic pollutants was proposed as one of the primary natural decomposition pathways. The screening and isolation of anaerobic/facultative PAHs‐degrading micro‐organisms also have remarkable achievements. Some enrichments or pure cultures of *Geobacter sulfurreducens, Desulfotomaculum*, *Achromobacter denitrificans*, etc. have been obtained using nitrate, sulphate, ferric iron or high valence manganese as the electron acceptor. Thus far, the initial steps of anaerobic PAHs degradation were identified as carboxylation or methylation, but the whole degradation process is still unclear (Zhang *et al*., 2021a, 2021b). Regarding future directions, more anaerobic and facultative micro‐organisms should be isolated to carry out a more systematic study on the anaerobic microbial degradation of PAHs via bioinformatics. For application, the anaerobic bioremediation technology has obvious competitive advantages, such as less disturbance of pollution and lower energy consumption. Moreover, for complex polluted environments such as soil and sediment, the *in‐situ* remediation technologies under natural conditions will attract wide attention, such as synergistic integration of aerobic, anaerobic and facultative micro‐organisms, *in‐situ* identification and regulation of functional microbial communities and activities. In the practice of contaminated soil remediation, environmental factors, such as soil pH, temperature, carbon source supply and properties of degraded compounds, greatly influence the effectiveness of PAHs bio‐remediation (Laothamteep *et al*., 2021). Therefore, more comprehensive research will be carried out on the anaerobic biodegradation of PAHs over the next 15 years. A major focus will be the metabolic pathways of microbial anaerobic degradation of PAHs, to reveal critical regulators and environmental variables for efficient anaerobic degradation. Besides, various methods for enhancing microbial remediation have been extensively reported, such as electro‐kinetic remediation, bio‐catalyst‐assisted remediation, bio‐surfactant flushing and nano‐remediation (Kuppusamy *et al*., 2017). In the near future, now bio‐augmentation technologies will significantly improve PAH biodegradation efficiency by micro‐organisms or enzymes in the natural environment.

In conclusion, the increasing public concerns of the environment has promoted the rapid development of new microbial technologies, focusing on solving environmental problems and maximizing the development of biological resources available to human beings. Many challenges and even more exciting breakthroughs are expected in the development of environmental microbial technology over the next 15 years.

## References

[mbt213924-bib-0001] Chengyue, L. , Yong, H. , and Hui, W. (2018) A new functional marker gene of polycyclic aromatic hydrocarbons (PAHs) degrading bacteria: pahE. Appl Environ Microbiol 85: e02399–e2418. 10.1128/AEM.02399-18 PMC6344622 30478232

[mbt213924-bib-0002] Conca, V. , Da Ros, C. , Valentino, F. , Eusebi, A.L. , Frison, N. , and Fatone, F. (2020) Long‐term validation of polyhydroxyalkanoates production potential from the sidestream of municipal wastewater treatment plant at pilot scale. Chem Eng J 390: 124627.

[mbt213924-bib-0003] Crognale, S. , Tonanzi, B. , Valentino, F. , Majone, M. , and Rossetti, S. (2019) Microbiome dynamics and phaC synthase genes selected in a pilot plant producing polyhydroxyalkanoate from the organic fraction of urban waste. Sci Total Environ 689: 765–773. 31280158 10.1016/j.scitotenv.2019.06.491

[mbt213924-bib-0004] De Donno Novelli, L. , Moreno Sayavedra, S. , and Rene, E.R. (2021) Polyhydroxyalkanoate (PHA) production via resource recovery from industrial waste streams: a review of techniques and perspectives. Biores Technol 331: 124985. 10.1016/j.biortech.2021.124985 33819906

[mbt213924-bib-0005] Kuppusamy, S. , Thavamani, P. , Venkateswarlu, K. , Lee, Y.B. , Naidu, R. , and Megharaj, M. (2017) Remediation approaches for polycyclic aromatic hydrocarbons (PAHs) contaminated soils: Technological constraints, emerging trends and future directions. Chemosphere 168: 944–968. 27823779 10.1016/j.chemosphere.2016.10.115

[mbt213924-bib-0006] Laothamteep, N. , Kawano, H. , Vejarano, F. , Suzuki‐Minakuchi, C. , Shintani, M. , Nojiri, H. , and Pinyakong, O. (2021) Effects of environmental factors and coexisting substrates on PAH degradation and transcriptomic responses of the defined bacterial consortium OPK. Environ Pollut 277: 116769. 33676341 10.1016/j.envpol.2021.116769

[mbt213924-bib-0007] Matos, M. , Cruz, R.A.P. , Cardoso, P. , Silva, F. , Freitas, E.B. , Carvalho, G. , and Reis, M.A.M. (2021) Combined strategies to boost polyhydroxyalkanoate production from fruit waste in a three‐stage pilot plant. ACS Sustainable Chemis Eng 9: 8270–8279.

[mbt213924-bib-0008] Moretto, G. , Russo, I. , Bolzonella, D. , Pavan, P. , Majone, M. , and Valentino, F. (2020) An urban biorefinery for food waste and biological sludge conversion into polyhydroxyalkanoates and biogas. Water Res 170: 115371. 31835138 10.1016/j.watres.2019.115371

[mbt213924-bib-0009] Nguyenhuynh, T. , Yoon, L.W. , Chow, Y.H. , and Chua, A.S.M. (2021) An insight into enrichment strategies for mixed culture in polyhydroxyalkanoate production: feedstocks, operating conditions and inherent challenges. Chem Eng J 420: 130488.

[mbt213924-bib-0010] Ryu, H. , Hahn, S. , Chang, Y. , and Chang, H. (2015) Production of poly(3‐hydroxybutyrate) by high cell density fed‐batch culture of *Alcaligenes eutrophus* with phospate limitation. Biotechnol Bioeng 55: 28–32. 10.1002/(SICI)1097-0290(19970705)55:1<28::AID-BIT4>3.0.CO;2-Z 18636441

[mbt213924-bib-0011] Zhang, Z. , Sun, J. , Guo, H. , Gong, X. , Wang, C. , and Wang, H. (2021a) Investigation of anaerobic biodegradation of phenanthrene by a sulfate‐dependent Geobacter sulfurreducens strain PheS2. J Hazard Mater 409: 124522. 33229262 10.1016/j.jhazmat.2020.124522

[mbt213924-bib-0012] Zhang, Z. , Sun, J. , Guo, H. , Wang, C. , Fang, T. , Rogers, M.J. , *et al*. (2021b) Anaerobic biodegradation of phenanthrene by a newly isolated nitrate‐dependent *Achromobacter denitrificans* strain PheN1 and exploration of the biotransformation processes by metabolite and genome analyses. Environ Microbiol 23: 908–923. 32812321 10.1111/1462-2920.15201

